# Is the Phytotoxic Effect of Digestive Fluid of *Nepenthes x ventrata* on Tomato Related to Reactive Oxygen Species?

**DOI:** 10.3390/plants12040755

**Published:** 2023-02-08

**Authors:** Pawel Staszek, Maciej Piekarniak, Agnieszka Wal, Urszula Krasuska, Agnieszka Gniazdowska

**Affiliations:** Department of Plant Physiology, Institute of Biology, Warsaw University of Life Sciences-SGGW, 02-776 Warsaw, Poland

**Keywords:** carnivorous plant, ROS, root growth, seed germination, allelopathy

## Abstract

The digestive fluid of pitcher plants is a rich source of enzymes and secondary metabolites, but its impact on higher plant growth and development remains unknown. The aim of the study was to determine the phytotoxicity of the digestive fluid of the pitcher plant (*Nepenthes x ventrata*) on the germination of tomato (*Solanum lycopersicum* L.) seeds, elongation growth and cell viability of roots of tomato seedlings. The digestive fluid was collected from pitchers before feeding and four days after feeding; the pH and electrical conductivity of the fluid were determined. Undiluted and 50% fluids were used in the study. An inhibition of germination of tomato seeds, by around 30% and 55%, was respectively observed in 50% and 100% digestive fluids collected before and after feeding. Digestive fluid did not affect the root growth of tomato seedlings; a slight (6%) inhibition was only observed after the application of 100% digestive fluid from an unfed trap. The roots of the tomato seedlings treated with undiluted fluid were characterized by reduced cell viability. Reactive oxygen species (H_2_O_2_ and O_2_^•−^) were mainly localized in the root apex regardless of the used phytotoxic cocktail, and did not differ in comparison to control plants.

## 1. Introduction

*Nepenthes x ventrata* belongs to the Nepenthaceae family and is a natural hybrid of *Nepenthes alata* Blanco and *Nepenthes ventricosa* Blanco. This species is endemic to forests in the Philippines [1]. *N. ventrata* forms jug-shaped containers that function as passive pitcher traps. Pitchers are filled with digestive fluid, being the mixture that enables the release of nutrients from the bodies of the captured prey [2].

Many enzymes have been found in the digestive fluid of pitcher plants: proteases, peptidases, phosphatases, esterases, ribonucleases and chitinases [3,4,5,6]. The aspartyl proteinases Nepenthesin-1 and Nepenthesin-2 have also been isolated from the digestive fluid [7]. The digestive fluid also contains a thaumatin-like protein with antibacterial and antifungal properties [8]. In addition, pitcher fluid contains compounds of low molecular weight, e.g., two naphthoquinones: droseron (3,5-dihydroxy-2-methyl-1,4-naphthoquinone) and its derivative 5-O-methyldroserone (2-methyl-3-hydroxy-5-methoxy-1,4- naphthoquinone) [9], of antifungal activity against a wide range of human and plant fungal pathogens [10]. Another low-molecular substance is plumbagin, isolated from *N. alata* [11] and characterized by antimalarial, antibacterial, antifungal and anticancer properties [12,13,14] and 7-methyl-juglone [11]. The digestive fluid of the Nepenthes species is rich in mineral nutrient composition (boron, calcium, copper, iron and nickel) and anions Cl^−^, Br^−^ and SO_4_^2−^ [11]. Prey digestion (feeding process) is accompanied by alteration in fluid composition, including reactive oxygen species (ROS) and reactive nitrogen species (RNS) levels [15].

The physicochemical properties of the digestive fluid, such as lower surface tension against water and high viscoelasticity, play an important role in preventing the escape of captured insects [16].

Application of allelopathic cocktails in cultivation can effectively influence weed populations and insect pests. Allelopathy thus offers an attractive environmentally friendly alternative to pesticides in agriculture [17]. Volatile organic compounds belonging to the group of allelochemicals could repel insects or mask the host’s odor to confuse them [18,19].

Digestive fluid as a mixture of many compounds with a possible phytotoxic potential may prove to be a key element in research on natural herbicides that would replace artificial and less specific compounds currently used in agriculture. The exact composition of the digestive fluid is unknown, but the digestive fluid of some species is known to contain compounds that have phytotoxic potential.

The aim of our study was to characterize some properties of phytotoxic cocktails and to determine the effect of the digestive fluid from pitchers of *N. ventrata*, collected before and after plant feeding, on tomato (*Solanum lycopersicum* L.) seed germination and root growth.

Based on the mode of action of many allelochemicals and phytotoxins [20], we hypothesized that pitcher plants’ digestive fluid impacts the seed germination and root growth of tomato seedlings by the induction of oxidative stress.

## 2. Results

### 2.1. Electrical Conductivity

The electrical conductivity and pH of the phytotoxic cocktail depended on the origin of the fluid (from fed or unfed traps) and the concentration. The highest pH value was found in distilled water used as a control, and the lowest in heat-treated, 100% fluid from fed traps (Table 1). The pH of the fluid from fed pitchers was lower than that from unfed ones, regardless of concentration or heat-treatment (Table 1).

The highest electrical conductivity (1.364 mS cm^−1^) was recorded for heat-treated undiluted fluid from unfed pitchers, and the lowest (0.0209 mS cm^−1^) for the distilled water (control) (Table 1). Digestive fluids (100%) were characterized by higher electrical conductivity compared to diluted ones, irrespective of the origin of the fluid and heat-treatment.

### 2.2. Germination Tests

Two-way Anova showed that there were no statistically significant differences between the germination of tomato seeds in crude fluid and heat-treated fluid (*p* = 0.73, F = 0.13). Due to the lack of associations, the dataset for crude and heat-treated fluids was not separated in the remaining analyses.

**Figure 1:**
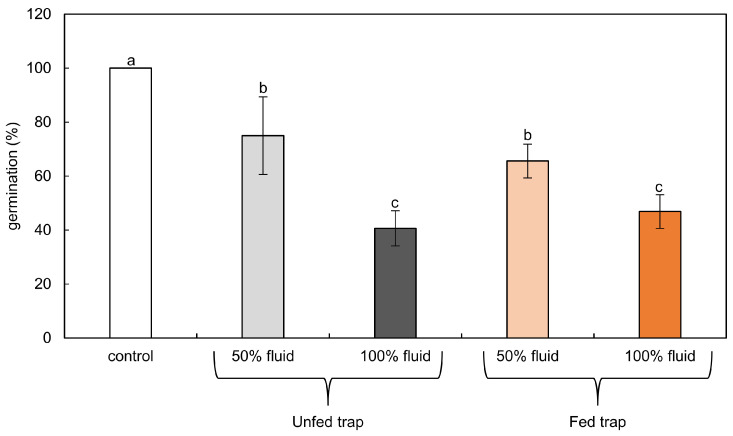
Germination of tomato seeds in water (control) or in phytotoxic cocktails of 100% or 50% digestive fluids from fed and unfed traps of pitcher plants. Due to the lack of significant differences, we did not separate data obtained after treatment with crude and heat-treaded fluids. Values are average ± SD of three replications. Homogenous groups (a–c) were evaluated using Fisher’s LSD post hoc test with *p* < 0.5.

Tomato seeds germinated in 100% fluid after three days of imbibition in water (Figure 1). Undiluted digestion fluids from unfed or fed traps inhibited the germination of tomato seeds by 59% or 53%, respectively. In diluted phytotoxic cocktails of fed and unfed pitchers, tomato seeds germinated in 67% and 75%, respectively (Figure 1).

### 2.3. Root Growth Biotest

Two-way Anova showed that there were no statistically significant differences between the growth of tomato seedlings in crude fluid and heat-treated fluid (*p* = 0.33, F = 0.95). Due to the lack of associations, the dataset for crude and heat-treated fluids was not separated in the remaining analyses.

**Figure 2:**
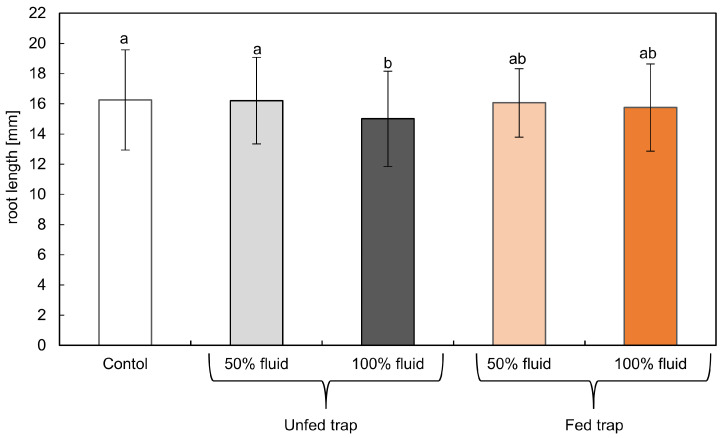
Length of roots of tomato seedlings growing for 24 h in water (control) or in phytotoxic cocktails of 100% or 50% digestive fluids from fed and unfed traps of pitcher plants. Due to the lack of significant differences, we did not separate data obtained after treatment, the division into crude and heat-treaded fluids was not included. Values are average ± SD of three replications. Homogenous groups (a, b) were evaluated using Fisher’s LSD post hoc test with *p* < 0.5.

Roots of the control tomato seedlings growing in water for 24 h were about 16 mm long. Fluids from traps of pitcher plants repressed the growth of tomato roots in only 6% (Figure 2). The longest roots were observed in seedlings treated with the diluted fluid from unfed traps. The strongest inhibition of root growth was noted for seedlings treated with 100% fluids from unfed traps. Root lengths measured in seedlings treated with diluted and undiluted fluid from fed traps differed by 0.25 mm and 0.5 mm from the control, respectively, but the differences were not statistically significant.

### 2.4. Test of Root Cell Viability

The viability of the root cells of seedlings treated for 24 h with crude and heat-treated phytotoxic cocktails slightly differed (Table 2). Excluding the control plants, the highest viability was estimated for roots of seedlings treated with crude, diluted fluid from fed traps, and the lowest viability was noticed for the roots of seedlings grown in 100% crude digestion fluid from unfed pitchers, which was very similar to 100% heat-treated fluid from fed traps (31,8–31,3% of damages, respectively). The roots of seedlings treated with 50% fluid were characterized by a higher viability than the roots of seedlings treated with undiluted fluid, irrespective of heat treatment.

### 2.5. In Situ H_2_O_2_ Localization in Tomato Seedlings Cultured

**Figure 3:**
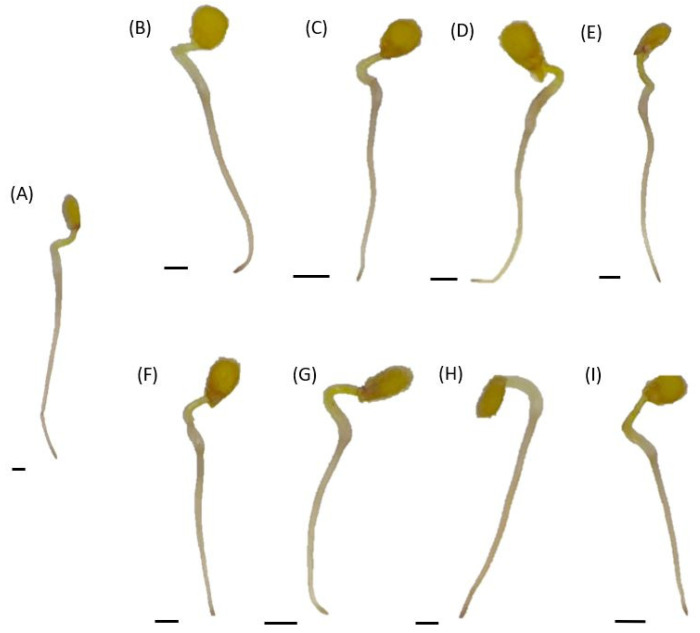
In situ H_2_O_2_ localization in tomato seedlings cultured for 24 h in water (the control) or in phytotoxic cocktails of 100% or 50% fed or unfed traps of pitcher plants. Control seedlings (**A**), seedlings growing in crude concentrated fluids of unfed traps (**B**), treated with crude diluted cocktail of unfed traps (**C**), cultured in crude concentrated fluids of fed traps (**D**), exposed to crude diluted cocktails of unfed traps (**E**), growing in heat-treated concentrated fluids of unfed traps (**F**), heat-treated diluted cocktails of unfed traps (**G**), heat-treated concentrated fluids of fed traps (**H**) and heat-treated diluted cocktails of fed traps (**I**). Bar = 2 mm.

H_2_O_2_ was localized using DAB staining as brown precipitates of polymerized DAB (Figure 3). For all tested seedlings, the most visible staining was detected in the root tips. Stronger coloration of the upper part of the roots, on the border with the hypocotyl, was observed in seedlings treated with heat-treated fluid, regardless of the concentration of the fluids or the feeding of the traps (from fed or unfed ones).

### 2.6. In Situ O_2_^•−^ Localization

**Figure 4:**
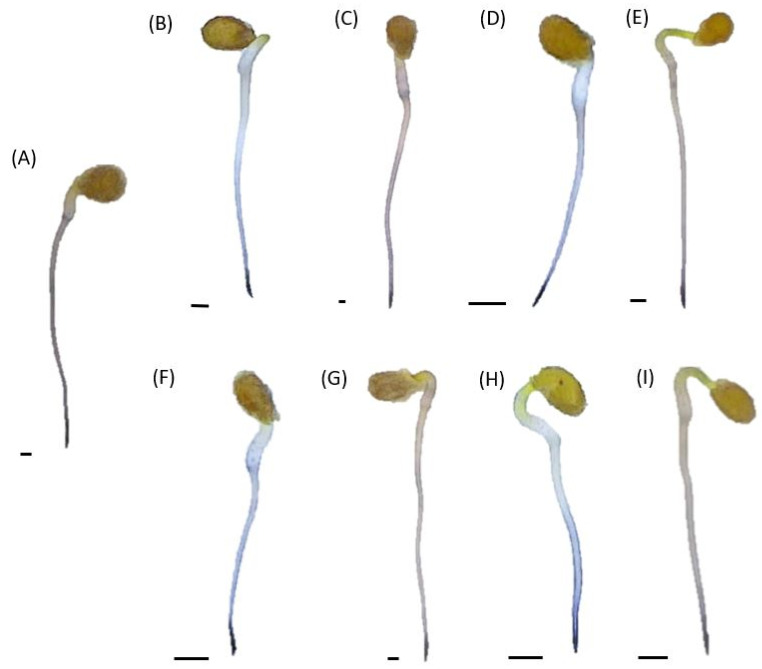
In situ O_2_^•−^ localization in tomato seedlings cultured for 24 h in water (control) or in phytotoxic cocktails of 100% or 50% fed or unfed traps of pitcher plants. Control seedlings (**A**), seedlings growing in crude concentrated fluids of unfed traps (**B**), treated with crude diluted cocktail of unfed traps (**C**), cultured in crude concentrated fluids of fed traps (**D**), exposed to crude diluted cocktails of unfed traps (**E**), growing in heat-treated concentrated fluids of unfed traps (**F**), heat-treated diluted cocktails of unfed traps (**G**), heat-treated concentrated fluids of fed traps (**H**) and heat-treated diluted cocktails of fed traps (**I**). Bar = 2 mm.

The histochemical localization of O_2_^•−^ was carried out using NBT staining. A dark blue coloration of formazan was observed in the root tips of all the tested seedlings (Figure 4). Only the outer layers of the roots were colored above the tips. The roots of seedlings treated with 50% digestive fluid were characterized by a slightly higher color intensity, regardless of the heating of the phytotoxic cocktails (Figure 4). However, in general, no differences in NBT staining were noticed between the roots of tomato seedlings treated with digestive fluids and those growing in water (Figure 4).

## 3. Discussion

*Nepenthes* is a plant which, by adapting to specific environmental conditions, in the process of evolution, developed a carnivorous syndrome. Although it is known where the components of the fluid are produced and how the substances resulting from the digestion of the prey are absorbed by the plant, knowledge about the composition of the digestive fluid is limited. The phenomenon of carnivorous syndrome in plants has been described for over 150 years; the isolation and identification of enzymes involved in digestion is still only fragmentary. It refers to both the composition of enzymes and other molecules in the digestive fluid. Their identification, structure and biosynthesis may be an interesting target for further research [9]. Until now, the digestive fluid of pitcher plants has been mostly examined in the context of plant nutrition, but its putative toxicity on neighboring plants should not be omitted. Our study is a unique observation of the impact of the digestive fluid on the germination and growth of other plants; acceptors, according to allelopathic terminology.

At the start of this study, we determined the impact of pitcher plant feeding and the inactivation of the fluid by heating on its phytotoxicity. No influence of the temperature inactivation on the toxicity of the digestive fluid on the germination of tomato seeds and the elongation growth of roots suggests temperature-resistant compounds of the fluid, rather than enzymes of the fluid, are responsible for its negative action. The feeding of the pitcher plant resulted in decreased pH of the fluid, which is a typical reaction in the digestion process [21], although it did not affect tomato seed germination. The germination of seeds was inhibited by phytotoxic cocktails with no respect to the pH of the fluid. Tomato seeds similarly germinated in the digestive fluid with a pH range of 3.3–3.8, taken from fed traps and in the digestive fluid of unfed traps (pH 5.5 to 6.1). The low pH of the digestive fluid also did not influence the elongation growth of the tomato roots. The pH value also seems to be correlated with bacterial community composition [22] and feeding. Taking to account no differences in cocktail phytotoxicity in the tomato seedlings before and after heating, it can be concluded that microorganisms from the fluid are not toxic to acceptor tissues; however, perhaps the experiments should be performed longer to observe such effects. The electrical conductivity of the digestive fluid showed no difference in dependence on feeding, nor heating; it only decreased as the dilution of the liquid was performed. Electrical conductivity in a range of 0.8–1.6 mS cm^−1^, observed in the research, indicates moderately saline soils, where only plants resistant to salinity can grow [23,24]. This data confirms the information that the pitcher fluid of *Nepenthes* plants (*N. alata*, *N. fusca*, *N.gracilis*, *N. mirabilis*, *N. superba*, *N. thorelii* and *N. ventricosa*) is a 25 mM KCl solution with few additional ions [11].

During the germination tests, solutions with a similar pH, but different digestive fluid concentration, were applied, on which the results of the experiment mainly depended. Undiluted digestive fluid, taken from a pitcher of *N. ventrata* fed with the proteins, inhibited tomato seed germination as compared to the 50% cocktail. Some compounds that may adversely affect the growth and development of other plants have been isolated from carnivorous plants. Studies conducted with the use of Portuguese sundew ((*Drosophyllum lusitanicum* L. (Link)) extracts have shown that the extracts inhibited the germination of lettuce (*Lactuca sativa* L.) and wheat (*Triticum aestivum* L.) seeds. Plumbagin has been identified as the main compound responsible for the inhibition of lettuce sprouting [25]. Other compounds, isolated from carnivorous plants, may show a toxic effect; these include two naphthoquinones, droserone and its derivative 5-O-methyldroserone, which have beem found in the digestive fluid of *N. khasiana* [10] or 7-methyl-juglone isolated from *N. alata* [11]. Previous reports from our laboratory indicated the presence of phenolic compounds in the digestive fluid, the total content of which increased in fed traps [15]. Phenolics are regarded as strong allelochemicals of various modes of action in acceptor plants [26].

Investigating the mode of action of allelopathic compounds is complicated due to the multitude of potential molecular targets. Biological tests using plants or plant tissues successfully detect the biological activity of many synthetic and natural compounds [27]. One of the first visible effects of phytotoxin’s action is the reduction of seed germination and/or seedling growth [28]. Diverse phytotoxins (like cyanamide, citral, coumarin) act as inhibitors of root tip cell division, influencing root growth [29,30,31]. Treatment of tomato seedlings with digestive fluids showed that components of these cocktails had no effect on the growth and development of the young seedlings. Morphology and length of the roots of tomato plants exposed to 100% and 50% digestive fluids of *N. ventrata* traps did not differ and were similar to those observed in control seedlings. It suggests that the impact of the phytotoxic cocktails of the digestive fluids on growth of the roots is weaker than on seed germination. This observation is atypical in allelopathy research, as most allelochemicals applied as the mixture or extract originated from the tissue of allelopathic plant are more toxic in growth tests than germination tests [32]. Many allelochemicals affect seed germination by inhibition of the activity of the enzymes crucial for the process. Extracts from the leaves and flowers of catnip (*Nepeta meyeri* Benth.) decreased α-amylase, ß-1,3 glucanase and protease activities in seeds of field dodder (*Cuscuta campestris* Yunck) [33]. Allelochemicals could disrupt the action of phytohormones in germinating seeds. The inhibition of germination of lettuce (*Lactuca sativa* L.) by myrigalone A, C-methylated dihydrochalcone from sweet gale (*Myrica gale* L.) was accompanied by alterations in gibberellins metabolism by inhibiting GA3 oxidase, as well as by interfering with the gibberellin signaling pathway [34]. Perturbations in ROS metabolism and the induction of oxidative stress were identified as the cause of inhibition of seed germination. Sunflower (*Helianthus annus* L.) extracts increased H_2_O_2_ content in mustard (*Sinapsis alba* L.) seeds, resulting in the restriction of their germination [35].

Although evidence for allelopathic interactions and/or the potential of allelochemicals is based on the evaluation of seed germination and seedling growth of the target species [28], these tests only provide general information regarding the biological effects of phytotoxins. They do not indicate a mechanism of their action [36,37]. In many studies, it has been demonstrated that, for allelopathy stress, as for other stresses, the induction of oxidative stress is the common response of acceptor plants [20,28,29,38]. Such observations are typical for both crude extracts of allelopathic plants, as well as for single allelopathic compounds. An accumulation of ROS, a modification of their localization and a disruption of cellular antioxidant systems were shown for various plants exposed to different allelochemicals (for review, see [20,29,38]. In our study, phytotoxic cocktails of digestive fluids did not lead to increased levels of H_2_O_2_ or O_2_^•−^. Moreover, no alteration in the localization of ROS in roots in response to digestive fluids were shown. It may be proposed that compounds in the fluids from *N. ventrata* traps do not impact ROS production. Although investigations of the activity of enzymes of the antioxidant system are still required, the presence of ROS and reactive nitrogen species in the digestion liquid of these plants has been recently revealed [15].

## 4. Materials and Methods

### 4.1. Experimental Model

In our experiments, we used tomato (*Solanum lycopersicum* L. cv Malinowy Ożarowski) seedlings as the test plant. A digestive fluid from pitchers of Nepenthes was applied in the tests as a phytotoxic cocktail.

Pitcher plants *Nepenthes x ventrata* Hort. ex Fleming [=(*N. ventricosa* Blanco × *N. alata* Blanco)] were grown in the greenhouse under conditions of constant high humidity (60%) and temperature (28 °C); light was supplemented with sodium lamps to obtain a 16-/8-h day/night photoperiod. A mixture of acid peat, perlite and sphagnum moss was used as a growing medium and plants were watered with distilled water every other day. The traps of pitcher plants, immediately after opening, were covered with gauze to avoid contamination by accidental insects. Digestive fluid was collected from fed or unfed mature pitchers (aerial type, length approximately 9 cm). Traps were fed with 40 μg hen egg white solution (1 µg µL^−1^) introduced with a pipette directly into a pitcher. Digestive fluids from fed traps were collected four days after feeding. The fluids (from fed and unfed pitchers) were shortly centrifuged (5522× *g*, 5 min at 4 °C), and the supernatants were transferred to the sterile tubes, frozen and used for biotests.

Before biotesting, digestive fluids from several traps were combined (fluid separately from fed and unfed traps) to maximize the volume of the phytotoxic cocktail. One portion of the fluid was boiled at 100 °C for 10 min (heat-treated fluid) and the rest was left untreated (crude fluid). Then, part of each fluid (heat-treated or crude) was diluted twice to obtain a 50% solution. The electrical conductivity and pH of phytotoxic cocktails were determined using a pH/conductivity meter (CPC-505 Elmetron, Zabrze, Poland, with suitable electrodes ECF-60 and EPS-1, respectively).

### 4.2. Germination Tests

Tomato seeds (*Solanum lycopersicum* L. cv. Malinowy Ożarowski) (commercially obtained from PNOS Sp. z o.o., Poland) were surface sterilized with 0.5% sodium hypochloride for 10 min at room temperature. Then, seeds were rinsed three times with distilled water, and 8 seeds were placed on Petri dishes (ø 3.5 cm) filled with filter paper wetted with 2.5 mL phytotoxic cocktail or 2.5 mL distilled water (control). The culture was carried out in a growth chamber (Fitotron Versatile Environmental Test Chamber-PAR-10Mn, by Sanyo, Osaka, Japan, model MLR-350H), in darkness at 20 °C for 3 days. Seeds were considered germinated when the radicle had emerged through the seed coat.

To investigate the impact of pitcher fluids on the growth of tomato seedlings, biotests were performed. Tomato seeds were germinated in water in darkness at 20 °C for 3–4 days. Then, 8 seedlings with roots of equal length (0.5 cm) were selected and transferred to Petri dishes (ø 3.5 cm) filled with filter paper wetted with 2.5 mL phytotoxic cocktails or 2.5 mL distilled water (control). Control seedlings and seedlings treated with digestive fluid were cultured in a growth chamber at 23/20 °C, 12-/12-h day/night regime, and a light intensity of 90 µmol PAR m^−2^ s^−1^ for 24 h as described by Krasuska et al. [39]. The root length of seedlings was measured after 24 h of the culture.

### 4.3. Test of Root Cell Viability

The viability of tomato root cells was determined using Evans blue staining [40]. The whole control seedlings or seedlings treated with pitcher fluids for 24 h were incubated in a 0.25% solution of Evans blue for 30 min at room temperature. Then, seedlings were washed three times in distilled water and roots were isolated, weighed and homogenized in 1 mL of a 1% solution of sodium dodecylsulphate (SDS). After 10 min centrifugation at 21,000× *g* at 4 °C, the supernatant was collected and absorbance was measured at 600 nm (Sunrise, Tecan, Männedorf, Switzerland). For the positive control (100% cell damage), the roots were boiled for 10 min, cooled and then treated as described above. Three roots were used for one repetition. The concentration of the extracted dye was estimated from the standard curve prepared with Evans blue in 1% SDS and expressed as mg g^−1^ FW or % of cell damage. The data were means of three measurements from each of three sets of experiments.

### 4.4. In Situ H_2_O_2_ Localization

The histochemical localization of H_2_O_2_ was carried out with 3,3′-diaminobenzidine (DAB) staining [39]. The roots of the control seedlings and seedlings treated with pitcher fluids for 24 h were washed twice in distilled water and incubated with DAB solution (1 mg mL^−1^) with 2 mM DMSO. Staining was carried out for 4 h at room temperature, in darkness.

### 4.5. In Situ O_2_^•−^ Localization

The histochemical localization of O_2_^•−^ was performed according to Beyer and Fridovich [41] using nitroblue tetrazolium (NBT) staining. Roots isolated from the control seedlings and seedlings treated with pitcher fluids for 24 h were washed twice in distilled water and incubated for 20 min in darkness at room temperature in 2 mM NBT (Sigma–Aldrich, Tokyo, Japan), freshly prepared in 10 mM Tris-HCl buffer pH 7.4 with 2 mM DMSO. After staining, the roots were cleaned for 24 h in chloral hydrate to avoid artifacts by removing oxidized phenolics [42]. O_2_^•−^ was visualized as a deposit of a dark blue insoluble formazan compound.

Images of seedlings stained with DAB and NBT were taken with the TAGARNO FHD TREND digital microscope (magnification x 5.2, F4 diaphragm, enhancement 10.2 dB, exposure time 1/15 s).

Data were obtained in at least three independent experiments with at least three repetitions each. The plants for experiments were randomly selected from 25 individuals. Data were analyzed using Statistica Software. Mean values were calculated and SD was provided. After Anova, homogenous groups were evaluated using Fisher’s LSD post hoc test.

## 5. Conclusions

We demonstrated the negative, dose-dependent effect of the digestive fluid of the pitcher plant on tomato seed germination; whereas its impact on root elongation was less evident, but still significant. Application of the digestive fluid decreased cell viability but did not alter ROS (H_2_O_2_ and O_2_^•−^) distribution in the roots of tomato seedlings. The explanation of the phytotoxic potential of the digestive fluid requires further study, in which the compounds of the trap fluid, being mainly secondary metabolites, are determined prior to their use in phytotoxicity tests. The context of perturbation in ROS levels in acceptor plants as the target of digestive fluid action needs examination of the activity of the enzymatic antioxidants in the tissue of the acceptor plants.

## Figures and Tables

**Table 1:** Electrical conductivity and pH of digestive fluid solutions used in the experiments as phytotoxic cocktails.

		pH	Electrical Conductivity [mS cm^−1^]
Control		6.29 ± 0.07 c	0.021 ± 0.017 A
Crude fluid			
Unfed trap	50% fluid	5.96 ± 0.17 bc	0.786 ± 0.078 B
	100% fluid	5.82 ± 0.03 b	1.333 ± 0.012 C
Fed trap	50% fluid	3.78 ± 0.19 a	0.704 ± 0.042 B
	100% fluid	3.38 ± 0.08 a	1.247 ± 0.017 C
Heat-treated fluid			
Unfed trap	50% fluid	6.09 ± 0.29 bc	0.771 ± 0.026 B
	100% fluid	5.54 ± 0.10 b	1.364 ± 0.052 C
Fed trap	50% fluid	3.83 ± 0.31 a	0.630 ± 0.040 B
	100% fluid	3.36 ± 0.16 a	1.127 ± 0.039 C

Table includes average values ± SD of three replicated experiments. Homogenous groups (a–c or A–C) were evaluated using Fisher’s LSD post hoc test with *p* < 0.5.

**Table 2:** Cell viability (Evans blue test) and cell damage of roots of tomato seedlings cultured in water or treated with digestive fluids from Nepenthes traps (crude, heat-treated, concentrated (100%) or diluted (50%)) after 24 h of the culture.

		Evans Blue Uptake to the Roots [mg dye g^−1^ FW]	Cell Damage [%]
Positive control (heat-treated roots)		0.476 ± 0.011 c	100 E
Control (seedlings grown in water)		0.086 ± 0.021 a	18.1 A
Crude fluid			
Unfed trap	50% fluid	0.122 ± 0.064 b	25.7 BC
	100% fluid	0.151 ± 0.053 b	31.8 D
Fed trap	50% fluid	0.100 ± 0.048 b	20.9 B
	100% fluid	0.140 ± 0.079 b	29.3 BC
Heat-treated fluid			
Unfed trap	50% fluid	0.109 ± 0.056 b	22.9 BC
	100% fluid	0.113 ± 0.055 b	23.7 D
Fed trap	50% fluid	0.135 ± 0.008 b	28.4 B
	100% fluid	0.149 ± 0.084 b	31.3 BC

Table includes average values ± SD of three replicated experiments. Homogenous groups (a–c or A–E) were evaluated using Fisher’s LSD post hoc test with *p* < 0.5.

## Data Availability

Upon request, the data will be provided by the corresponding author.
